# Activation of Systemic Inflammation and Oxidative Stress in Adolescent Girls with Polycystic Ovary Syndrome in Combination with Metabolic Disorders and Excessive Body Weight

**DOI:** 10.3390/jcm9051399

**Published:** 2020-05-09

**Authors:** Elena Khashchenko, Mikhail Vysokikh, Elena Uvarova, Lyubov Krechetova, Valentina Vtorushina, Tatyana Ivanets, Maria Volodina, Nadezhda Tarasova, Iuliia Sukhanova, Gennady Sukhikh

**Affiliations:** 1 2nd Gynecological Department for Children and Adolescents, FSBI “National Medical Research Center for Obstetrics, Gynecology and Perinatology Named After Academician V.I. Kulakov” Ministry of Healthcare of the Russian Federation, 117997 Moscow, Russia; e_uvarova@oparina4.ru; 2 Laboratory of Mitochondrial Medicine, FSBI “National Medical Research Center for Obstetrics, Gynecology and Perinatology Named After Academician V.I. Kulakov” Ministry of Healthcare of the Russian Federation, 117997 Moscow, Russia; mvolodina@hse.ru (M.V.); compasstar@gmail.com (N.T.); suhanova_julia@hotmail.com (I.S.); 3 A.N. Belozersky Research Institute of Physico-Chemical Biology, 119992 Moscow, Russia; 4 Laboratory of Clinical Immunology, FSBI “National Medical Research Center for Obstetrics, Gynecology and Perinatology Named After Academician V.I. Kulakov” Ministry of Healthcare of the Russian Federation, 117997 Moscow, Russia; l_krechetova@oparina4.ru (L.K.); v_vtoruchina@oparina4.ru (V.V.); 5 Clinical Diagnostic Laboratory, FSBI “National Medical Research Center for Obstetrics, Gynecology and Perinatology Named After Academician V.I. Kulakov” Ministry of Healthcare of the Russian Federation, 117997 Moscow, Russia; t_ivanets@oparina4.ru; 6 Institute of Cognitive Neuroscience, Centre for Bioelectric Interfaces, NRU HSE, Myasnitskaya 13, Building 4, 109028 Moscow, Russia; 7 Institute of Molecular Medicine, Sechenov First Moscow State Medical University, Trubetskaya str. 8, bld. 2, 119991 Moscow, Russia; 8 FSBI “National Medical Research Center for Obstetrics, Gynecology and Perinatology Named After Academician V.I. Kulakov” Ministry of Healthcare of the Russian Federation, 117997 Moscow, Russia; g_sukhikh@oparina4.ru

**Keywords:** polycystic ovary syndrome, insulin resistance, systemic inflammation, mitochondrial dysfunction, interleukins, C-reactive protein, lipid peroxidation, malondialdehyde, apoptosis

## Abstract

Relevance: Mitochondrial dysfunction and systemic inflammation are believed to play pivotal role in the pathogenesis of polycystic ovary syndrome (PCOS) and related complications of metabolic disorders in adult patients. Though such researches are limited or almost absent in adolescents. The aim of the study is to evaluate the impact of mitochondrial dysfunction and systemic inflammation on PCOS pathogenesis during adolescence with regard to body mass index and insulin resistance. Design: a case-control study. Methods: The study included 95 adolescent girls (15 to 17 years old inclusive) diagnosed with PCOS based on the Rotterdam criteria. The control group consisted of 30 healthy girls of the same age with a regular menstrual cycle. All participants were subjected to a full clinical and instrumental examination, as well as an assessment of the levels of leptin, C-reactive protein (CRP), and malondialdehyde (MDA) as oxidative stress marker. Serum levels of IL-6, IL-10, IL-18, TNF-α, and plasma concentrations of macrophage migration inhibitory factor (MIF), sFas, and sFasL were determined. Patients with PCOS were divided into groups according to the presence of metabolic disorders (MD) (impaired glucose tolerance and/or over insulin resistance) and normal weight or excessive weight (NW or OW). Results: Patients with PCOS of NW in the absence of metabolic disorders (MD−/NW) had a lower concentration of MDA and a higher level of IL-10 compared to healthy girls (*p* < 0.05). The group (MD−/NW) was characterized with lower levels of CRP, leptin, MDA, and higher levels of sFasL, when compared to OW patients with PCOS in the absence of metabolic disorders (MD−/OW) (*p* < 0.05). Overweight adolescent girls with PCOS and metabolic disorders (MD+/OW) showed higher CRP, leptin, and a two-fold increase in IL-6 and IL-18 concentrations compared to the control group of healthy girls (*p* < 0.05 for all parameters). The group (MD+/OW) was also characterized with higher levels of CRP, leptin, MDA, IL-18, MIF (*p* < 0.05), when compared to overweight patients with PCOS in the absence of metabolic disorders (MD−/NW). In comparison with the MD−/OW group, the obese insulin resistant girls with PCOS (MD+/OW) had a highera level of IL-18 (*p* < 0.05). Moreover, the MD+/OW girls demonstrated a significant increase in CRP, MDA and IL-18 levels when compared to the MD+/NW group (*p* < 0.05). OW girls with PCOS without MD (MD−/OW) had lower concentrations of sFasL compared to healthy girls (*p* < 0.05), and higher levels of MDA compared to MD+/NW (*p* < 0.05). Adolescent girls of NW with PCOS and with MD (MD+/NW) had lower levels of MDA compared to the control group of healthy girls (*p* < 0.05). These data are confirmed by a correlation analysis and two-factor ANOVA test. Conclusions: Lean girls with PCOS demonstrate the protective mechanism of decrease in oxidative stress mediated by the activation of antioxidant defense, reduction of lipid peroxidation and systemic inflammation. Excessive weight and metabolic disorders in adolescents with PCOS are the most significant factors in reducing the capacity of antioxidant systems, activation of oxidative stress, mitochondrial dysfunction, and systemic inflammation.

## 1. Introduction

Polycystic ovary syndrome (PCOS) is a chronic syndrome, that most frequently manifests in adolescence and observed in 3% to 30% cases [1]. PCOS is usually associated with multiple metabolic disorders (MD) that change with age, from insulin resistance (IR) in adolescents, to dyslipidemia, obesity (especially visceral), arterial hypertension, fatty hepatosis, type 2 diabetes, and an increased risk of myocardial infarction that usually occur in adulthood [2,3,4]. The incidence of metabolic disorders among adolescent PCOS girls is 33%, which is 3–5 times higher compared to healthy females of the same age and body mass index (BMI) [5]. Although the co-occurrence of IR and obesity is highly prevalent, they separately contribute to the pathogenesis of PCOS and occur independently of one another [1,2].

The etiology of PCOS among adolescent girls is not fully understood. There is a growing number of publications nowadays that demonstrate the relationship between chronic systemic inflammation and mitochondrial dysfunction as well as their significance in the etiology of PCOS and its long-term consequences [2,6,7]. It was found that the development of PCOS among patients of reproductive age is accompanied by systemic increase of oxidative stress that occurs because of mitochondrial dysfunction and reduced antioxidant protection [7,8,9]. It has been shown that, compared to healthy women of reproductive age, PCOS patients have increased blood levels of lymphocyte and monocyte counts, as well as elevated levels of C-reactive protein (CRP), proinflammatory cytokines (tumor necrosis factor-α (TNF- α), interleukins (IL-1, IL-6, IL-18)) and high levels of lipid peroxidation, protein carbonylation products and advanced oxidation protein products [10,11].

It is known that the initial activation of the systemic inflammatory response observed in plasma in patients with PCOS is associated with an increased production of reactive oxygen species (ROS) because of mitochondrial damage and dysfunction, which in turn plays a key role in the development of oxidative stress and further vicious cycle activation of inflammatory response. Excessive ROS production leads to a compensatory increase in the consumption of non-enzymatic antioxidants and an increase in antioxidant enzymes activity [12]. Compared to healthy women, PCOS patients of reproductive age, regardless of BMI, had lower concentrations of reduced glutathione and peroxyredoxines in peripheral blood, and increased activity of glutathione peroxidase (GPX), superoxide dismutase (SOD), and catalase enzymes activities [6,7,8].

The above described data were obtained mainly during studies on adult women with a long history of PCOS and MD [13]. However, PCOS manifests during puberty. We propose the severity of systemic inflammatory response and mitochondrial dysfunction in adolescent uncomplicated PCOS at the initial stages of the disease could be less pronounced than in adults.

Thus, it is possible that adolescent girls with PCOS would have a less severe systemic inflammatory response and mitochondrial dysfunction. The confirmation of this hypothesis could become a basis for the development of preventive therapy that would impede progression of the disease and its complications.

Aim of the current study: to characterize systemic inflammatory response and oxidative stress hallmarks among adolescent girls with PCOS, presented with or without increased BMI and/or MD.

## 2. Materials and Methods

Total of 95 girls were included in the study, aged 15 to 17 years, presenting complete Rotterdam PCOS diagnostic criteria (oligo-/amenorrhea; clinical and/or biochemical signs of hyperandrogenism; polycystic ovaries detected by ultrasound). The additional inclusion criteria were: The onset of menarche at least 2 years prior; the absence of other endocrine diseases; absence of medication for at least 3 months prior to the study; informed consent of the patient and her legal representative for participation in the research study. The exclusion criteria were: Pelvic tumors; an exacerbated state of a chronic and acute somatic and/or infectious disease; mental illnesses; genetic syndromes and malformations. The control group consisted of 30 healthy adolescent girls of the same age with regular menstruation and no gynecological and endocrine pathologies.

General clinical examination was performed for all participants. It included a detailed medical history, gathering of anthropometric data (height, BMI, waist to hip circumference ratio), and assessment of hypertrichosis severity. BMI was considered as normal if the values were less than 25 kg/m^2+^ (normal weight, NW) and excessive if values were over 25 kg/m^2^ (overweight, OW).

Concentrations of total protein, uric acid, creatinine, direct and total bilirubin, glucose, Ca^2+^, Fe^2+/3+^, and highly sensitive CRP in venous blood were determined for all participants. The lipid profile was assessed by total cholesterol, triglycerides (TG), low-density lipoproteins (LDL), high-density lipoproteins (HDL), and atherogenic index. Analyses were performed on automated biochemical analyzers BA-400 and A-25 using photometric and turbidimetric methods and reagents from Biosystems (Spain, Barcelona). An oral glucose tolerance test (OGTT) was performed 12–16 hours after the last meal. Glucose and immunoreactive insulin (IRI) levels were measured in venous blood on an empty stomach, and the second evaluation 120 minutes after taking 75 g of glucose. The homeostasis model assessment of insulin resistance (HOMA-IR) was calculated. The visceral adiposity index (VAI) was used to indirectly estimate the volume of abdominal adipose tissue:

VAI = (WC ÷ (36.58 + (1.89 × BMI)) × (TG ÷ 0.81) × (1.52 ÷ HDL)

where WC (waist circumference) is measured in cm, BMI—body mass index in kg/m^2^, TG and HDL in mmol/L.

On the 3rd to 5th day of a spontaneous or gestagen-induced menstrual cycle, all girls included in the study were subjected to a pelvic ultrasound examination and an extended analysis of the blood hormonal profile: luteinizing hormone, follicle-stimulating hormone, estradiol, testosterone, sex hormone binding globulin, prolactin, cortisol, androstenedione, dehydroepiandrosterone sulfate, 17-OH-progesterone, thyroxine, thyroid-stimulating hormone. 

Serum concentrations of IL-6, IL-10, IL-18, and TNF-α were measured by enzyme-linked immunosorbent assays (ELISA) using test systems from eBioscience (San Diego, CA, USA) in the laboratory of clinical immunology.

To estimate the oxidative stress, the level of malondialdehyde (MDA), a marker of lipid peroxidation (LPO), was measured in plasma using a modified Jentzsch method. This method is based on the reaction of MDA with 2-thiobarbituric acid that results in the formation of a colored complex with a maximum absorption at 535 nm [14,15].

Concentrations of leptin, macrophage migration inhibitory factor (MIF), markers of apoptosis (the receptor sFas and its ligand sFasL) were measured using the multiplex assay kit (Milliplex MAP Magnetic Bead Panel 1, Merck KGaA, Darmstadt, Germany) in blood plasma samples using a Luminex 200 flow analyzer (Luminex, Austin, TX, USA) according to the manufacturer’s instructions. 

Statistical data analysis was performed in MS Excel and Statistica 8. Comparison of variables with a normal distribution was performed by analysis of variance (ANOVA). The set of groups was compared in pairs by post-hoc method of a posteriori multiple comparisons considering Fisher’s least significant difference (least significant difference test, LSD). Parameters that did not fit normal distribution were analyzed using Kruskal–Wallis rank tests. Subsequently, the intergroup differences were assessed by post-hoc test with ranking according to the Dunn or Siegel-Castellan criterion. Correlations were estimated using Spearman’s rank correlation coefficient. To evaluate the impact of categorical factors on dependent variables the multifactorial analysis (factorial ANOVA) was performed.

The study was conducted in accordance with the Declaration of Helsinki, and the protocol was approved by the Ethics Committee for Biomedical Research at the National Medical Research Center for Obstetrics, Gynecology and Perinatology named after Academician V.I. Kulakov” Ministry of Healthcare of the Russian Federation, Moscow, Russia (Project identification code № 13, 06 December 2016).

## 3. Results

The study shows that 58 of the 95 (61.1%) girls with PCOS had normal OGTT and HOMA-IR index, whereas 37 (38.9 %) of the teenagers had impaired glucose tolerance and/or insulin resistance (HOMA-IR ≥ 3.46 c.u.). Based on these data, first group of girls was assigned to a cohort without metabolic disorders (MD−), and the second group—to the cohort with metabolic disorders (MD+). In the second cohort (MD+), 15 of the 37 (40.5%) girls had both impaired OGT and IR, 14 (37.8%) had OGT only and 8 (21.6%) girls had IR only. BMI analysis in the cohorts demonstrated that the majority of the girls (48, 82.8%)) in the MD− group had normal BMI (<25 kg/m^2^, normal weight (NW)), while only 13 (35.1%) of the girls in the MD+ group had normal BMI values. The girls with PCOS with excessive weight (*n* = 34) were referred to the overweight groups (OW, BMI values were over 25 kg/m^2^) (see Table 1). The control group (30 girls) had no MD and had normal values of HOMA-IR and BMI.

For the analysis of effects of independent variables, MD and BMI, all patients were divided into four groups depending on BMI value and the presence/absence of MD (see Table 1).

Blood chemistry analyses showed significant differences between the 1st (MD−/NW) and control groups. The 1st group had higher total protein (73.2 ± 5.3 vs. 70.6 ± 4.6 g/L, *p* = 0.0431) and direct bilirubin (4.5 ± 2.0 vs. 3.1 ± 1.0 μM, *p* = 0.0251) levels. At the same time, the only difference between the first and the third (MD+/NW) groups was a statistically significant higher blood iron level (23.8 ± 8.0 vs. 16.6 ± 6.8 μM, *p* = 0.0423). Compared to the control group, the third group had higher levels of total and direct bilirubin (19.0 ± 5.5 vs. 11.1 ± 4.2 μM, *p* = 0.0076 and 5.1 ± 3.6 vs. 3.1 ± 1.0 μM, *p* = 0.0155, respectively) and iron (23.8 ± 8.0 vs. 16.6 ± 6.8 μM, *p* = 0.0343).

Blood lipid profile analysis did not reveal significant differences in the studied parameters (Table 1) between the 1st (MD−/NW) and control groups. The 3rd group (MD+/NW) had higher TG (*p* = 0.0056), higher atherogenic index (AI, *p* = 0.0262) and lower HDL levels (*p* = 0.0391) compared to the first group (MD−/NW). These differences along with higher WC (73.5 ± 8.2 vs. 68.2 ± 6.2 cm; *p* = 0.0302) and BMI (60.9 ± 6.6 vs. 56.5 ± 5.5 kg/m^2^) cause an upward tendency of VAI in the third group (MD+/NW) compared to the first group (MD−/NW). Moreover, girls in the 3rd group had higher AI values compared to the control group (*p* = 0.0206).

Compared to the control group, girls from the 4th group (MD+/OW) had significantly higher values of HOMA-IR (*p* = 0.0026) and fasting glucose (*p* = 0.0021), but also higher levels of TG (*p* = 0.0237), AI (*p* = 0.0010) and lower HDL levels (*p* = 0.0459) and increased risk of cardiovascular disease according to the VAI index (*p* = 0.0105). As shown in Table 1, similar differences were found between the 4th and first (MD−/NW) groups.

Studies performed in the last three years demonstrate the role of excessive adipose tissue in chronic low-grade systemic inflammatory response, which affects functioning of multiple organs and leads to the activation of the immune system [16,17].

No significant differences in complete blood count (CBC) were found between patients with PCOS and the control group: CBC was within the normal reference range. Intergroup comparison using Dunn’s test showed that the 4th group (MD+/OW) had significantly higher amount of leukocytes (7.9 (6.9–8.0) vs. 6.1 (5.3–6.6) 10^9^/L, *p* = 0.0235), platelets (321.0 (281.0–331.0) vs. 240.0 (213.5–278.0) 10^9^/L, *p* = 0.0012), neutrophils (55.0% (48.6–62.0) vs. 46.6% (41.3–55.3), *p* = 0.0440), and higher erythrocyte sedimentation rate (5.0 (4.0–10.0) vs. 2.0 (2.0–3.0) mm/h, *p* = 0.0036) compared to the 1st group (MD−/NW). These increased CBC values may indicate the presence of low-grade inflammation in PCOS girls with excessive weight and MD.

CRP level was estimated as one of the most sensitive marker of systemic inflammation [16]. We demonstrate that the group of OW girls with PCOS had higher levels of CRP compared to other groups of girls with normal BMI values with (*p* = 0.0127) or without (*p* = 0.0226) MD (Table 2). Moreover, adolescents of the 4th group (MD+/OW) had significantly higher level of CRP compared to the 1st group (MD−/NW) (*p* = 0.0011) and the control group (*p* = 0.0239).

In our study on adolescent girls, we did not find either PCOS or MD to be an independent factor of increased levels of CRP. According to the correlation (r = 0.29; *p* < 0.05) and two-factor analysis (*p* = 0.0028), a significant direct (independent) effect was confirmed only for high BMI. Nevertheless, the combination of factors VAI ≥ 1 and MD show significant effect on CRP level (F = 8.63; *p* = 0.0043, ANOVA), that prove marked activation of systemic inflammation in the presence of visceral adiposity and IR in adolescent girls with PCOS. 

According to our data, hyperleptinemia was observed in patients with PCOS with increased BMI and MD, which was expected because leptin is secreted mainly by subcutaneous fat tissue (Table 2). Moreover, in comparison with the 1st group (MD−/NW), the level of this proinflammatory adipokine was significantly higher in overweight patients with (*p* = 0.0267) and without (*p* = 0.0004) MD. The leptin level was also significantly increased in the 4th group (MD+/OW) compared to the control group (*p* = 0.0034). Two-factor analysis of variance (ANOVA) identified high BMI (*p* = 0.0007) and metabolic disorders (*p* = 0.0083) as independent factors of increased levels of leptin in the blood, which is regarded as a marker of activation of the systemic inflammatory response in PCOS complicated by overweight and metabolic disorders.

Thus, our data show that PCOS itself is not associated with low-grade inflammation in adolescent girls, included in the study. An increased level of inflammatory markers (leukocyte level, CRP, leptin) was detected in adolescents with high BMI, VAI, and/or MD. It is important to point out, that in obese PCOS group without IR, the levels of CRP and leptin were significantly higher, than in controls, that proves the activation of adipocytes and chronic inflammation even in the absence of MD but because of excessive weight in PCOS patients already in adolescence.

These findings were corroborated by the study of the cytokine profile in peripheral blood in the girls with PCOS (Table 3). The 1st group (MD−/NW) had higher concentration of anti-inflammatory cytokine IL-10 (*p* = 0.0165) compared to the control group. The level of IL-10 in the 3rd group (MD+/NW) did not differ significantly compared to the 1st group or to the control group.

Patients with PCOS of 4th group (MD+/OW) had twice as high concentration of proinflammatory cytokines IL-6 (*p* = 0.0314) and IL-18 (*p* = 0.0292) compared to the control group. In addition, these patients had the highest concentration of IL-18 among the compared groups (see Table 3). Significantly higher concentration of proinflammatory cytokine MIF (*p* = 0.0465) in the 4th group, compared to the 1st (MD+/NW), also indicates the activation of systemic inflammation in the 4th group.

Patients with PCOS had different levels of anti- and proapoptotic markers sFas and sFasL depending on the BMI and the presence of MD (Table 3). As it is known, the Fas and Fas ligand (FasL) system plays a key role in the regulation of programmed cell death. Fas is a membrane protein receptor that exists in membrane-bound or soluble forms. Interaction between the transmembrane Fas receptor and its ligand (sFasL) triggers an apoptotic cascade, while soluble sFas inhibits Fas-mediated apoptosis, thus preventing signal transmission [18]. Our results show that the concentration of the antiapoptotic marker sFas was higher in patients with PCOS and normal BMI compared to the girls in the control group (3.3 ± 5.8 ng/mL and 2.1 ± 0.8 ng/mL; *p* = 0.0302, Mann–Whitney test). Moreover, patients of the 1st group (MD−/NW) had the highest level of proapoptotic factor sFasL among all groups of PCOS patients and was significantly different from the 2nd group (MD−/OW) (*p* = 0.0399). In contrast, the 2nd group had the lowest sFasL concentration which was significantly different from the control group (*p* = 0.0099).

Two-factor analysis of variance (ANOVA) demonstrated that MD had an independent positive impact on the IL-18 concentration (*p* = 0.0137) and a negative impact on the sFas/sFasL ratio (*p* = 0.0163). The BMI factor also had a negative impact on the sFas/sFasL ratio (*p* = 0.0207). In addition, interaction of these factors was significant for proinflammatory cytokines MIF (*p* = 0.0228) and IL-18 (*p* = 0.0259). The BMI factor did not affect levels of cytokines in the absence of MD. However, the presence of both factors (high BMI and MD) was associated with a two-fold increase in the level of proinflammatory cytokines MIF (*p* = 0.0228) and IL-18 and with significantly reduced anti-apoptotic defense. These results confirm that there is an activation of systemic inflammation and apoptosis in adolescent girls with PCOS, high BMI, and MD.

Thus, adolescent girls with PCOS and normal BMI and without MD had alterations in the cytokine profile of peripheral blood due to a higher concentration of anti-inflammatory (IL-10) and antiapoptotic factors. On the contrary, high BMI and/or MD, and especially the combination of these two factors in adolescent girls with PCOS, was associated with the activation of a systemic inflammatory response. Such activation of low-grade inflammation is described in studies of patients with PCOS of reproductive age, regardless of BMI or the presence of MD [9,16].

The analysis of the markers of oxidative stress among adolescent girls with PCOS also revealed patterns not typical for adult women (Table 4). First, patients of the 1st group (МD−/NW) had a lower concentration of a marker of lipid peroxidation (LPO), malondialdehyde (MDA) (*p* = 0.0271), which indicates a reduction in oxidative stress level. Girls with PCOS of the 3rd group (MD+/NW), compared to healthy girls, also had a lower level of LPO as evidenced by a lower level of MDA (*p* = 0.0174). Contrary to that, girls with high BMI demonstrated activation of LPO, according to significantly higher MDA level in the blood of the girls in the 2nd group (MD−/OW) compared to the 3rd group (MD+/NW) (*p* = 0.0336), and in girls of the 4th group (MD+/OW) compared to the 3rd (MD+/NW) (*p* = 0.0005) and 1st groups (MD−/NW) (*p* = 0.0002).

**Figure 1:**
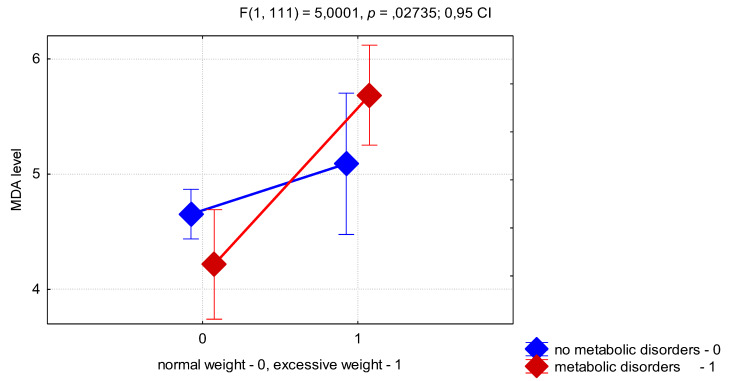
Two-factor analysis of variance of the interaction between BMI and metabolic disorders (MD) on the MDA level in adolescent girls with PCOS (LS means, vertical bars denote 0.55 confidence intervals).

To assess the effect of high BMI and presence of MD on MDA levels we performed a two-factor ANOVA. This test showed that BMI had an independent effect on the MDA level (*p* < 0.0001). Also we found that combination of factors VAI ≥ 1 and MD show significant effect on MDA level (F = 4.91; *p* = 0.0289, ANOVA), that prove pronounce activation of LPO in the presence of visceral adiposity and IR in adolescent girls with PCOS. In addition, the interaction of factors of excessive BMI and the presence of MD was highly significant for the MDA level (*p* = 0.0273) (Figure 1). Thus, as can be seen from Figure 1, the presence of MD in OW girls was associated with a higher level of MDA than in the MD−/OW group. Unexpectedly, the presence of MD in girls with normal BMI was associated with a lower level of MDA than in the MD−/NW group, which can be explained by a compensatory mechanism for the regulation of oxidative stress, presented in adolescence.

Also, we demonstrated a positive correlation between BMI and the severity of systemic inflammation in patients with PCOS, as judged by higher CRP levels in peripheral blood (r = 0.29; *p* < 0.05). Elevated CRP levels in adolescents with PCOS are associated with MDA accumulation (r = 0.45; *p* < 0.05). No such correlations were found in the group of healthy girls. 

The study allowed us to identify positive correlations between blood cytokine levels and oxidative stress markers, which are the most prominent among patients of the 4th group (MD+/OW). For instance, for MDA levels we found a positive correlation with proinflammatory cytokine MIF levels (r = 0.65, *p* < 0.05), and a negative correlation with the levels of antiapoptotic marker sFas (r = −0.53, *p* < 0.05). In the overall group of patients with PCOS (*n* = 95), similar correlations were found, for example, between MDA and IL-6 levels (r = 0.33, *p* < 0.05) and between TNF-α and leptin levels (r = 0.28, *p* < 0.05). The above-mentioned correlations were not significant in the control group. Thus, a positive correlation between systemic inflammation and oxidative stress hallmarks is typical for adolescent girls with PCOS complicated by high BMI and MD.

## 4. Discussion

It is well-known that alterations in lipid profile and higher values of HOMA-IR are characteristic in women with PCOS of reproductive age regardless of BMI [1,2]. Our data suggest that the independent effect of BMI on lipid profile in girls with PCOS is less. However, the presence of a combination of high BMI and MD already in adolescence leads to the development of severe dyslipidemia, increased atherogenicity and visceral adiposity, that result in a higher risk of cardiovascular diseases.

According to the available data from the literature and meta-analyses, adult patients with PCOS have a higher level of CRP not due to obesity, rather due to the presence of the disease itself [16,17]. Our results failed to prove the independent impact of either PCOS or metabolic disorders on CRP levels in adolescent girls. Nevertheless, the factors of high BMI and interrelation of VAI≥1 and presence of MD show significant effect on CRP level, that highlight activation of systemic inflammation in adolescent girls with PCOS complicated with the visceral adiposity and IR.

From the results of our study, it can be concluded that the observed multidirectional changes in the systemic inflammatory response and oxidative stress in adolescent girls with PCOS have different pathogenesis in patients of normal or increased BMI, with or without metabolic disorders. Our findings suggest that patients with PCOS, normal BMI, and without MD may have a compensatory adaptive mechanism to maintain low levels of LPO and counteract systemic inflammatory response. A possible protective mechanism could be the depletion of NADPH pools in heme synthesis and degradation processes. Indirect confirmation of the bilirubin-biliverdin cycle acting as a protective antioxidant mechanism in PCOS patients is the blood chemistry profile. We demonstrated that PCOS patients with normal BMI had significantly higher bilirubin level in the blood compared to healthy girls. It is known that biliverdin, a product of bilirubin metabolism, is one of the key intracellular antioxidants. So, the relative increase in bilirubin content in patients with PCOS and normal weight can be considered as a protective adaptive mechanism against oxidative stress activation and the pro-inflammatory status observed in PCOS patients. 

On the other hand, mitochondrial control of oxidative stress and the switching of the cell strategy to the depletion of NADPH pools in the cycles of heme synthesis and degradation restricts the use of this energy substrate by the NADPH oxidase enzyme of immune cells. The implementation of this protective antioxidant mechanism may cause a decrease in markers of systemic inflammatory response in PCOS girls with normal BMI in adolescence, which has been shown in our work. According to the literature, it may also play a role in the increased predisposition of adolescent girls to nonspecific infectious and somatic diseases [19].

As is known regarding the physiological levels of LPO and ROS products, a delicate balance between pro- and antioxidants at local and system levels is necessary for the development and functioning of all cellular systems [20]. In particular, it has been shown that the physiological levels of ROS is necessary for the induction of cell proliferation and differentiation, follicle growth and maturation, as well as for ovulation [21,22]. The parallel maintenance of the physiological levels of ROS and high-energy adenosine triphosphate (ATP) molecules, the mitochondrial membrane potential and the level of intact mitochondrial DNA is necessary to trigger and maintain cell proliferation processes [23]. In vitro studies have shown the activation of HIF-1 transcription factor (hypoxia induced factor-1) and the initiation of cell division after the restoration of normal ROS levels, NAD+/NADH ratio and mitochondrial membrane potential [23]. To date, in vitro cultures and animal models have shown that excessively low ROS levels in the ovaries along with an increased concentration of antioxidants has a negative impact on the maturation and fertilization of the follicle [21,22]. On the other hand, excessive ROS production, which exceeds antioxidant protection capacity, can trigger apoptosis and induce ovarian cell damage [8,20]. As observed in our work, a decrease in LPO levels in patients with PCOS with normal BMI and without MD, compared to the levels typical for healthy girls, could contribute to impaired folliculogenesis and to anovulation in this group of patients.

At the same time, metabolic disorders in overweight women lead to dyslipidemia along with fatty acid accumulation and reduction of glucose utilization. It is known that the accumulation of free fatty acids (FFA) in tissues, besides its direct toxic effect, causes an increase in the level of phospholipid peroxidation, that could cause an increase in mitochondrial membrane permeability and mitochondrial dysfunction, and triggers cascades of reactions leading to apoptosis [24]. In addition, FFA have a direct uncoupling effect on the processes of respiration and synthesis of high-energy molecules in the mitochondria, thereby reducing the mitochondrial membrane potential. Because of the common origin of mitochondria with bacteria, damaged proteins of the mitochondrial matrix, components of mitochondrial membranes, and molecules of mitochondrial DNA, which get into the bloodstream are considered as foreign agents (Damage Associated Molecular Patterns, DAMP) by the immune cells [25]. Ultimately, that leads to activation of the immune response [20,24,25]. The results of our study show oxidative stress and inflammatory response activation in overweight girls with PCOS and even more pronounce increase in combination of excessive weight and metabolic disorders. We suggest that the oxidative stress and activation of systemic inflammation in the overweight PCOS groups is associated with dyslipidemia and cholesterol excess, and accumulation of fatty acids instead of glucose utilization. As we know from literature data, uncoupling effect of FFA lowering mitochondrial transmembrane potential could lead to opening of the permeability transition pore, inflammasome activation and DAMPs increase, thus resulting in systemic inflammation activation, ROS production and lipid peroxidation, that we see in overweight and PCOS group with MD. Moreover, the activation of the systemic inflammatory response in overweight PCOS girls with MD may also be due to the ability of FFA to indirectly activate NADPH oxidase and induce the production of ROS by immune cells and adipocytes. FFA can also activate the signaling pathway of proinflammatory factor NF-KB (nuclear factor kB) and induce the production of proinflammatory cytokines (IL-1, IL-6, TNF-α) by mononuclear cells and white adipose tissue cells in patients with PCOS of reproductive age [26,27]. In addition, the increased level of glucose in circulation is a prerequisite to increased oxidation of glucose in the pentosophosphate pathway in mononuclear cells of girls with PCOS, IR and hyperglycemia, that is also associated with activation of the NF-KB signaling pathway and an increased level of proinflammatory cytokines IL-6, TNF-α, and ROS production by immune cells [2]. It is important that the above-described mechanisms contribute to the chronic systemic inflammation are observed not only in overweight women with PCOS, but also in adult PCOS patients with normal weight and without MD [9,16,26,27]. We found increased levels of CRP and leptin in overweight PCOS adolescent group even in the absence of IR. The results of factor analysis evidenced the importance of MD, excessive weight and visceral adiposity in the activation of systemic inflammation in girls with PCOS. Inflammatory activation in OW/MD+ PCOS patients was evidenced by higher levels of proinflammatory cytokines IL-18, IL-6, and MIF. On the contrary PCOS NW/MD-group was characterized with higher level of anti-inflammatory IL-10. Multiple factor analysis demonstrates significant inflammatory activation according to the levels of IL-18 and MIF in combination of excessive weight, MD, or high VAI. The observed proinflammatory cytokine profile in blood in majority of cases caused by neutrophils activation, higher ROS and peroxide production, and related endothelial cells activation is indirectly proved with significantly higher level of MIF in PCOS OW/MD+ group. 

Thus, the results of our study show that the severity of systemic inflammatory response and mitochondrial dysfunction in adolescent uncomplicated PCOS at the initial stages of the disease are less pronounced than in the presence of excessive weight and MD. The difference of systemic inflammatory activation and mitochondrial dysfunction depending on the presence of excessive BMI and MD in adolescent girls with PCOS is not typical for adult patients with PCOS according to the literary data [6,7,8,16,17].Thus, we did not observe an activation of systemic inflammation and oxidative stress in adolescent girls with PCOS and normative BMI and the absence of MD. On the contrary, in this group of PCOS patients there was a significant increase in the concentration of anti-inflammatory cytokine IL-10 and high antiapoptotic protection sFas/sFasL along with a decrease in LPO, apparently because of the homeostasis control system and adaptive compensative mechanism of antioxidant defense in adolescence. However, the suppression of ROS levels and oxidative stress in this group of girls can cause, among other things, a disruption of cell-to-cell interactions that is necessary for the growth and development of the follicle and ovulation and lead to polycystic phenotype of ovaries observed in PCOS [21,23].

Overweight girls with PCOS especially complicated with metabolic disorders demonstrate an activation of oxidative stress and systemic inflammation, and a reduction of antiapoptotic defense systems already in adolescence, which is also typical for adult patients with PCOS, apparently because of the failure of homeostasis and adaptation. It is important to point out that excessive BMI, especially in combination with impaired carbohydrate metabolism, is a factor of exacerbating insulin resistance, the development of dyslipidemia, procoagulant and pro-inflammatory activation, increasing atherogenicity, visceral adiposity and the risk of systemic endothelial dysfunction in PCOS patients already in adolescence. Of note, there is a lack of such studies among adolescent patients with clinical and laboratory criteria for PCOS. Understanding the fundamental mechanisms of PCOS development at the onset of the disease will allow to develop therapeutic approaches to prevent further disease progression.

## 5. Conclusions

An adaptive mechanism is activated to counteract the manifestation of oxidative stress and systemic inflammation in adolescent girls with PCOS of normal weight. Overweight adolescent girls with PCOS have manifestations of oxidative stress due to impaired metabolism of cholesterol and glucose, which is further enhanced if metabolic disorders are present. The activation of the systemic inflammatory response is associated with oxidative stress and decreased antiapoptotic defense in overweight adolescent girls with PCOS and insulin resistance.

Clinical implication of uncovering the fundamental mechanisms of PCOS development with regard to body weight and metabolic disorders consists in a possibility of potential mitochondria-oriented therapy of this pathology.

Examination of girls with PCOS, in addition to BMI, include waist circumference, blood lipid profile, the atherogenic coefficient (CA = (total cholesterol − HDL)/HDL), and the visceral adiposity index (VAI) should be calculated as an integral marker of the morphofunctional state of visceral adipose tissue and insulin secretion. There is an increased risk of endothelial dysfunction and cardiovascular complications if VAI > 1, that is important already in adolescence. The presence of impaired carbohydrate metabolism and insulin resistance should be determined for all patients with PCOS in adolescence, including patients with normative body weight. In order to identify pro-inflammatory activation the level of C-reactive protein and leptin are useful.

Because of the different pathogenic mechanisms of mitochondrial dysfunction in patients with different metabolic phenotypes of PCOS, adolescents with PCOS and normal BMI in the absence of metabolic disorders could be managed with mitochondrial-oriented therapy to maintain a balance of ROS production in the respiratory cycle of mitochondria (e.g., lipoic acid, inositols).

Adolescents with PCOS, increased BMI, and impaired carbohydrate metabolism in addition to a restrictive diet and physical exercises, could be managed with biguanides or insulin sensitizers in combination with supplements that improve mitochondrial functioning and reduce the manifestations of oxidative stress and systemic inflammation (e.g., L-carnitine, lipoic acid, inositols).

## Figures and Tables

**Table 1:** Blood lipid profile values, results of the oral glucose tolerance test (OGTT) and visceral adiposity index (VAI) values in adolescents with polycystic ovary syndrome (PCOS), grouped by BMI and presence of MD.

Values	Groups of PCOS Patients (*n* = 95)				Сontrol Group (*n* = 30)	*p*-Value
	MD−/NW (*n* = 48)	МD−/OW (*n* = 10)	МD+/NW (*n* = 13)	МD+/OW (*n* = 24)		
Cholesterol **, mmol/L	4.2 (3.9–5.1)	4.4 (4.0–5.1)	4.9 (4.5–5.2)	4.0 (3.7–4.9)	4.1 (3.5–4.8)	0.4879
TG *, mmol/L	0.7 ± 0.2	0.9 ± 0.5	1.1 ± 0.5	1.2 ± 0.5	0.8 ± 0.6	0.0001 1:3 = 0.0056 1:4 < 0.0001 4:5 = 0.0237
HDL *, mmol/L	1.6 ± 0.4	1.5 ± 0.2	1.4 ± 0.6	1.4 ± 0.5	1.7 ± 0.4	0.0180 1:3 = 0.0391 4:5 = 0.0459
LDL *, mmol/L	2.1 ± 0.7	2.2 ± 0.5	2.6 ± 0.7	2.4 ± 0.8	2.0 ± 0.7	0.1802 1:4 = 0.0363
Atherogenic index *	1.8 ± 0.6	2.1 ± 0.4	2.6 ± 0.7	2.8 ± 1.3	1.7 ± 0.7	0.0046 1:3 = 0.0262 1:4 = 0.0016 3:5 = 0.0206 4:5 = 0.0010
Glucose 0’ *, mmol/L	5.0 ± 0.4	4.9 ± 0.4	5.2 ± 0.5	5.2 ± 0.5	4.9 ± 0.4	0.0626 1:4 = 0.0257 2:4 = 0.0427 4:5 = 0.0021
Glucose 120’ *, mmol/L	5.3 ± 0.9	5.9 ± 0.6	6.6 ± 1.3	7.9 ± 3.7	-	0.0009 1:4 = 0.0001 2:4 = 0.0180
Insulin 0’ *, mIU/mL	9.7 ± 3.1	12.4 ± 4.0	16.3 ± 9.0	30.1 ± 26.6	10.5 ± 3.3	0.0000 1:4 < 0.0001 2:4 = 0.0019 3:4 = 0.0080 4:5 = 0.0010
Insulin 120’ **, mIU/mL	20.0 (7.4–29.3)	17.0 (8.4–20.7)	22.0 (15.0–40.9)	59.2 (37.7–129.2)	-	0.0000 1:4 < 0.0001 2:4 = 0.0002 3:4 = 0.0019
НОМА-IR **	1.9 (1.5–2.8)	2.9 (1.9–3.4)	3.6 (2.0–4.4)	5.4 (3.8–7.6)	2.2 (1.9–2.5)	0.0000 1:4 < 0.0001 2:4 = 0.0391 4:5 = 0.0026
VAI **	0.6 (0.5–1.0)	0.9 (0.6–1.7)	1.0 (0.8–1.6)	1.6 (1.1–2.0)	0.6 (0.4–1.0)	0.0013 1:4 = 0.0014 4:5 = 0.0105

* variable with a normal distribution, data are presented as mean ± standard deviation, ANOVA test (LSD test and Newman–Keuls test for intergroup comparisons); ** variable with a non-normal distribution, data are presented as median, 25–75 percentiles (Kruskal–Wallis test and Dunn test for intergroup comparisons).

**Table 2:** The level of CRP and leptin in peripheral blood in adolescent girls with PCOS, grouped by BMI and presence of MD.

Parameters	Groups of PCOS Patients (*n* = 95)				Control Group (*n* = 30)	*p*-Value
	MD−/NW (*n* = 48)	MD−/OW (*n* = 10)	MD +/NW (*n* = 13)	MD +/OW (*n* = 24)		
CRP *, mg/L	0.7 ± 0.7	1.8 ± 1.8	0.8 ± 1.2	2.2 ± 2.4	1.0 ± 0.9	0.0412 1:2 = 0.0226 1:4 = 0.0011 3:4 = 0.0127 4:5 = 0.0239
Leptin **, ng/mL	17.4 (8.9–24.6)	51.7 (23.7–65.7)	35.2 (29.2–43.4)	50.7 (43.6–63.4)	19.9 (12.4–28.5)	<0.0001 1:2 = 0.0267 1:4 = 0.0004 4:5 = 0.0034

* variable with a normal distribution, data are presented as mean ± standard deviation, ANOVA test (LSD test and Newman–Keuls test for intergroup comparisons); ** variable with a non-normal distribution, data are presented as median, 25–75 percentiles (Kruskal-Wallis test and Dunn test for intergroup comparisons).

**Table 3:** Cytokine profile of peripheral blood in adolescent girls with PCOS, grouped by BMI and presence of MD.

Cytokines	Groups of PCOS Patients (*n* = 95)				Control Group (*n* = 30)	*p*-Value
	MD−/NW (*n* = 48)	MD−/OW (*n* = 10)	MD +/NW (*n* = 13)	MD + /OW (*n* = 24)		
IL-6 **, pg/mL	0.06 (0.05–0.54)	0.30 (0.07–0.41)	0.06 (0.06–0.34)	0.62 (0.24–1.20)	0.39 (0.05–0.86)	0.5390 4:5 = 0.0314
IL-10 *, pg/mL	1.03 ± 0.68	0.76 ± 0.63	0.78 ± 0.36	0.91 ± 0.45	0.62 ± 0.35	0.5180 1:5 = 0.0165
IL-18 *, pg/mL	80.54 ± 80.23	65.55 ± 65.82	78.26 ± 49.57	158.15 ± 138.00	68.54 ± 63.08	0.0407 1:4 = 0.0078 2:4 = 0.0199 3:4 = 0.0282 4:5 = 0.0292
TNFα **, pg/mL	8.72 (6.23–9.68)	7.78 (6.75–9.05)	7.07 (6.44–8.38)	8.74 (6.68–10.67)	7.91 (7.18–8.94)	0.7308
MIF *, ng/mL	1.26 ± 0.74	1.29 ± 0.70	1.31 ± 1.06	2.61 ± 3.11	1.84 ± 2.51	0.2988 1:4 = 0.0465
sFas **, ng/mL	2.20 (2.06–2.55)	2.59 (1.95–2.68)	2.17 (1.89–2.32)	2.30 (2.05–2.52)	1.98 (1.78–2.46)	0.6564
sFasL *, pg/mL	2.48 ± 1.45	1.07 ± 0.64	1.71 ± 1.29	1.89 ± 1.16	2.88 ± 1.67	0.0694 1:2 = 0.0399 2:5 = 0.0099
sFas/sFasL *, pg/mL	3046.0 ± 4792.4	1549.9 ± 760.1	2719.0 ± 3202.6	2070.1 ± 2656.0	1163.5 ± 759.6	0.1465

* variable with a normal distribution, data are presented as mean ± standard deviation, ANOVA test (LSD test for cross-group comparisons); ** variable with a non-normal distribution, data are presented as median, 25–75 percentile, Kruskal–Wallis test (Dunn’s criterion for intergroup comparisons).

**Table 4:** Malondialdehyde (MDA) level in peripheral blood in adolescent girls with PCOS, grouped by BMI and metabolic disorders.

Parameter	Groups of PCOS Patients (*n* = 95)				Control Group (*n* = 30)	*p*-Value
	МD−/NW (*n* = 48)	МD−/OW (*n* = 10)	МD+/NW (*n* = 13)	МD+/OW (*n* = 24)		
Plasma MDA*, μМ	4.44 ± 1.07	4.99 ± 0.88	4.13 ± 0.85	5.40 ± 1.01	4.98 ± 0.66	0.0005 1:4 = 0.0002 1:5 = 0.0271 2:3 = 0.0336 3:4 = 0.0005 3:5 = 0.0174

* data are presented as mean ± standard deviation, ANOVA test (LSD test for intergroup comparisons).
